# Genetic Variants in Vitamin D Pathway Genes and Risk of Pancreas Cancer; Results from a Population-Based Case-Control Study in Ontario, Canada

**DOI:** 10.1371/journal.pone.0066768

**Published:** 2013-06-24

**Authors:** Laura N. Anderson, Michelle Cotterchio, Julia A. Knight, Ayelet Borgida, Steven Gallinger, Sean P. Cleary

**Affiliations:** 1 Samuel Lunenfeld Research Institute, Mount Sinai Hospital, Toronto, Ontario, Canada; 2 Dalla Lana School of Public Health, University of Toronto, Toronto, Ontario, Canada; 3 Cancer Prevention and Control, Cancer Care Ontario, Toronto, Ontario, Canada; 4 Zane Cohen Digestive Diseases Clinical Research Centre, Mount Sinai Hospital, Toronto, Ontario, Canada; 5 Department of Surgery, University of Toronto, Toronto, Ontario, Canada; MOE Key Laboratory of Environment and Health, School of Public Health, Tongji Medical College, Huazhong University of Science and Technology, China

## Abstract

Recent studies of 25-hydroxyvitamin D (25(OH)D) levels and pancreas cancer have suggested a potential role of the vitamin D pathway in the etiology of this fatal disease. Variants in vitamin-D related genes are known to affect 25(OH)D levels and function and it is unknown if these variants may influence pancreatic cancer risk. The association between 87 single nucleotide polymorphisms (SNPs) in 11 genes was evaluated within the Ontario Pancreas Cancer Study, a population-based case-control study. Pancreatic cancer cases with pathology confirmed adenocarcinoma were identified from the Ontario Cancer Registry (n = 628) and controls were identified through random digit dialing (n = 1193). Age and sex adjusted odds ratios (OR) and 95% confidence intervals (CI) were estimated by multivariate logistic regression. SNPs in the *CYP24A1*, *CYP2R1*, calcium sensing receptor (*CASR*), vitamin D binding protein (*GC*), retinoid X receptor-alpha (*RXRA*) and megalin (*LRP2*) genes were significantly associated with pancreas cancer risk. For example, pancreas cancer risk was inversely associated with *CYP2R1* rs10741657 (AA versus GG, OR = 0.70; 95%CI: 0.51–0.95) and positively with *CYP24A1* rs6127119 (TT versus CC. OR = 1.94; 95%CI: 1.28–2.94). None of the associations were statistically significant after adjustment for multiple comparisons. Vitamin D pathway gene variants may be associated with pancreas cancer risk and future studies are needed to understand the possible role of vitamin D in tumorigenesis and may have implications for cancer-prevention strategies.

## Introduction

Pancreas cancer has a poor prognosis with a five-year survival rate of less than 6%. The few well-established risk factors for pancreas cancer include family history, smoking, and obesity [1] with few actionable targets for disease prevention. Genetic studies have identified rare highly-penetrant mutations in certain genes but the genetic basis of the majority of pancreatic cancer is unknown [2]. Recent genome-wide association studies (GWAS) have identified several loci associated with pancreas cancer risk, including one at chromosome 13q22.1 that has been shown in both European and Chinese populations [3], [4].

Vitamin D, from diet and sun exposure, has been associated with reduced risk of several cancers, including colon, prostate and breast [5], [6], [7], [8]. Laboratory studies provide support for biologic mechanisms explaining how vitamin D may reduce cancer risk [9], [10], [11]; as a result, there has been considerable interest in vitamin D as a cancer-prevention strategy. The association between vitamin D and pancreas cancer is conflicting with the recent publication of two large pooled studies; one suggesting increased risk of pancreas cancer associated with high levels of circulating 25-hydroxyvitamin D (25(OH)D) [12] and the other suggesting an inverse association between 25(OH)D and pancreas cancer risk [13]. Studies of dietary vitamin D intake [14], [15] and predicted vitamin D levels [16], [17] and pancreatic cancer risk have yielded inconsistent results.

Several genes are involved in vitamin D activity, and GWAS have identified polymorphisms significantly associated with 25(OH)D concentrations [18], [19]. Heritability estimates for 25(OH)D range from 28–77% [20], [21], [22]. Vitamin D related genetic variants have been investigated in relation to the risk of other cancers, including prostate, breast, and colon with inconclusive results [23]. Since genetic variation in vitamin D related genes influence long-term serum vitamin D levels and several variants have been independently associated with other malignancies, we hypothesized that variation within vitamin D related genes may be associated with pancreatic cancer. Since no previous studies have evaluated variants in vitamin D metabolism genes and pancreas cancer risk we used data from the Ontario Pancreas Cancer Study [24] to evaluate these associations.

## Materials and Methods

### Ethics Statement

This study was approved by the Research Ethics Boards of the University Health Network and Mount Sinai Hospital, Toronto, Canada.

### Study Design

Data for this study were collected as part of the Ontario Pancreas Cancer Study, a population-based case-control study. The Ontario Pancreas Cancer Study is a member of the Pancreatic Cancer Case-Control Consortium (PanC4) and is one of seven sites which contribute to the Pancreatic Cancer Genetic Epidemiology Consortium (PACGENE) [24], [25].

### Recruitment of Cases and Controls

Pathology-confirmed pancreas cancer cases with a first confirmed adenocarcinoma of the pancreas or adenocarcinoma metastasis in the province of Ontario were identified from the Ontario Cancer Registry from 2002–2009 using rapid case ascertainment. Cases with neuroendocrine tumors and other non-adenocaricoma histologies were excluded from the study. Eighteen study participants had a family member in the study and 9 of these cases were randomly excluded from each family to eliminate related individuals. Population-based controls were recruited as part of the Ontario Familial Colorectal Cancer Registry (OFCCR) through random-digit dialing methods and the Ministry of Finance Property Assessment Database during 2002–2003. Controls had no personal history of pancreas or colorectal cancer.

### Data Collection

Cases and controls completed mailed self-administered Personal History Questionnaires that collected information on a range of topics including medical history and lifestyle factors. Established pancreas cancer risk factors, including smoking, body mass index (BMI) and family history of pancreas cancer, have been associated with increased pancreas cancer risk in this study [24]. Blood was collected from both cases and controls. DNA was isolated from lymphocytes using phenol-chloroform extraction or spin columns (Qiagen, Valencia, CA) and stored at 4°C.

### Candidate Gene and SNP Selection

We selected candidate vitamin D pathway genes from the extensive vitamin D literature and two recent GWAS [18], [19]. Functional candidate SNPs within these genes were selected for inclusion and additional tagging SNPs were identified using HapMap phase 3 release 2 data. Tag SNPs were selected for each gene using the pairwise selection method in the CEU population and specifying minor allele frequency (MAF)>10% and linkage disequilibrium (LD) R^2^<0.80; candidate SNPs identified a priori were specified as inclusion criteria in the tag SNP selection. In total, 87 SNPs in 11 genes (Table 1) were included in this study. Genotyping was conducted at the Clinical Genomics Centre (Toronto, Canada) using the MassARRAY® iPLEX Gold Sequenom Platform (Sequenom, USA; www.sequenom.com). Genotypes were analyzed using the Sequenom MassArray Typer v3.4 software and visual assessment of the data was used for confirmation. Ten study participants were excluded as genotyping failed for >10% of SNPs. All plates included positive and negative controls and 10% of samples were genotyped in duplicate as internal controls. The percent agreement was >95% for all duplicates.

**Table 1:** List of vitamin D pathway genes, official gene symbols and number of polymorphisms genotyped.

Gene Name	Gene symbol	Number of genotyped polymorphisms
Calcium sensing receptor	*CASR*	13
Cubilin	*CUBN*	1
cytochrome P450, family 24, subfamily A, polypeptide 1	*CYP24A1*	18
cytochrome P450, family 27, subfamily B, polypeptide 1	*CYP27B1*	3
cytochrome P450, family 2, subfamily R, polypeptide 1	*CYP2R1*	5
7-dehydrocholesterol reductase	*DHCR7*	2
Group-specific component (vitamin D binding protein)	*GC*	5
Low density lipoprotein receptor-related protein 2 (megalin)	*LRP2*	12
NAD synthetase 1	*NADSYN1*	2
Retinoid X recptor, alpha	*RXRA*	6
Vitamin D (1, 25-dihydroxyvitamin D3) receptor	*VDR*	20

### Statistical Analysis

Age and sex adjusted odds ratios (OR) and 95% confidence intervals (CI) were estimated using logistic regression. We evaluated the impact of excluding non-Caucasian study participants from the analysis; however, there were no substantial differences in the effect estimates and therefore the results are presented for all study participants combined. All statistical analyses were conducted using R version 2.14.1 and Hardy Weinberg equilibrium (HWE) was evaluated using the R Genetics package [26]. All statistical tests were two-sided with a statistical significance level of p<0.05.

This study was approved by the Research Ethics Boards of the University Health Network and Mount Sinai Hospital, Toronto, Canada.

## Results

Table 2 describes the study population. Controls were age and sex matched to cases and the mean age of both cases and controls was 64 years and 52% were male. Among the controls 94% were Caucasian versus 84% of cases. Both BMI and family history of pancreas cancer were significantly associated with increased pancreas cancer risk. The MAF and test for HWE among controls only, are shown in Table 3 for each SNP; significant departure from HWE (P<0.05) was detected for four VDR SNPs (rs1989969, rs2238136, rs2238135, and rs2853564) and one CYP2R1 SNP (rs11023374) and this did not change when the analyses were restricted to Caucasians only; therefore, we excluded these five SNPs from all subsequent analyses.

**Table 2:** Distribution of pancreatic cancer cases, controls and odds ratios for selected subject characteristics.

Variable	Cases (n = 628)	Controls (n = 1193)	Age & Sex adjusted Odds Ratio
	Mean (SD)	Mean (SD)	
Age	64.3 (10.2) Range 20–89	63.6 (8.9) Range 29–79	N/A
	No. (%)	No. (%)	
Sex			
Male	329 (52%)	621 (52%)	N/A
Female	299 (48%)	572 (48%)	
Ethnicity			
Caucasian	530 (84%)	1123 (94%)	1.00
Non-Caucasian	94 (15%)	48 (4%)	4.24 (2.96–6.15)
BMI (categorical)			
<25.0	209 (33%)	487 (41%)	1.00
25.0–29.9	244 (39%)	486 (41%)	1.17 (0.93–1.47)
≥30.0	156 (25%)	209 (18%)	1.76 (1.35–2.30)
Family history of pancreas cancer in 1^st^ or 2^nd^ degree relative			
No	530 (84%)	1156 (97%)	1.00
Yes	98 (16%)	37 (3%)	5.75 (3.92–8.62)

**Table 3:** Associations between 87 SNPs in vitamin D-related genes and pancreas cancer risk among Ontario cases (n = 628) and controls (n = 1193) and age and sex adjusted OR using a log additive model

Gene	SNP	Minor Allele	Major Allele	MAF^a^	HWE p-value^b^	Age and Sex adjusted OR^c^	p-value	Adj p-value^d^
*CASR*	rs1042636	G	A	8.76	0.278	1.09	0.490	0.867
*CASR*	rs12485716	A	G	27.7	0.148	0.89	0.152	0.481
*CASR*	rs1354162	A	C	10.41	0.277	1.02	0.876	0.963
*CASR*	rs1501900	T	A	21.21	1.000	0.95	0.589	0.890
*CASR*	rs1801725	T	G	14.19	0.475	1.06	0.562	0.889
*CASR*	rs2134221	C	T	33.01	0.358	1.03	0.695	0.945
*CASR*	rs3804592	A	G	14.33	0.156	0.81	**0.043**	0.425
*CASR*	rs3845918	A	G	26.49	0.766	1.00	0.983	0.994
*CASR*	rs4678172	A	C	27.75	0.220	1.04	0.613	0.890
*CASR*	rs4678174	C	T	31.35	0.060	0.90	0.149	0.481
*CASR*	rs6438705	A	G	18.15	0.119	1.05	0.619	0.890
*CASR*	rs6762782	A	G	39.68	0.164	1.00	0.977	0.994
*CASR*	rs7432045	C	T	21.34	0.862	0.97	0.711	0.945
*CUBN*	rs1907362	A	G	3.91	1.000	1.16	0.399	0.789
*CYP24A1*	rs1570669	G	A	33.7	0.796	1.09	0.266	0.648
*CYP24A1*	rs2181874	A	G	24.98	0.354	1.12	0.148	0.481
*CYP24A1*	rs2209314	C	T	25.65	0.544	1.05	0.560	0.889
*CYP24A1*	rs2245153	C	T	18.83	0.924	0.98	0.782	0.963
*CYP24A1*	rs2248461	A	G	37.05	0.069	1.02	0.801	0.963
*CYP24A1*	rs2296241	G	A	47.32	0.202	1.07	0.336	0.731
*CYP24A1*	rs2426498	G	C	13.45	0.534	0.99	0.897	0.963
*CYP24A1*	rs2585428	A	G	46.55	0.449	0.91	0.154	0.481
*CYP24A1*	rs2762941	A	G	38.41	0.245	1.14	0.070	0.425
*CYP24A1*	rs4809955	G	A	13.55	0.804	1.16	0.145	0.481
*CYP24A1*	rs4809957	G	A	21.15	0.098	1.17	0.063	0.425
*CYP24A1*	rs4809958	G	T	15.17	0.071	1.23	**0.026**	0.425
*CYP24A1*	rs4809959	A	G	49.71	0.385	1.05	0.437	0.809
*CYP24A1*	rs6013897	A	T	20.79	1.000	1.02	0.788	0.963
*CYP24A1*	rs6013905	C	T	15.3	0.093	1.21	**0.040**	0.425
*CYP24A1*	rs6022999	G	A	23.65	0.810	0.95	0.504	0.867
*CYP24A1*	rs6097805	G	A	24.94	0.164	1.15	0.072	0.425
*CYP24A1*	rs6127119	T	C	22.2	0.179	1.20	**0.027**	0.425
*CYP27B1*	rs10877012	T	G	32.69	0.742	1.04	0.634	0.890
*CYP27B1*	rs4646536	C	T	33.11	0.744	1.07	0.360	0.764
*CYP27B1*	rs703842	C	T	33.18	0.696	1.06	0.399	0.789
*CYP2R1*	rs10741657	A	G	39.04	0.301	0.85	**0.026**	0.425
*CYP2R1*	rs11023374	C	T	27.21	0.002	1.16	**0.050**	0.425
*CYP2R1*	rs11819875	G	T	18.61	0.849	0.98	0.853	0.963
*CYP2R1*	rs12794714	A	G	43.08	0.140	1.16	0.031	0.425
*CYP2R1*	rs2060793	A	G	39.14	0.302	0.86	**0.035**	0.425
*DHCR7*	rs1630498	G	T	22.02	0.399	0.92	**0.308**	0.703
*DHCR7*	rs1790349	G	A	15.55	0.583	1.00	0.995	0.995
*GC*	rs1491711	C	G	34.42	0.083	1.11	0.138	0.481
*GC*	rs1491718	C	T	9.72	0.743	0.85	0.203	0.519
*GC*	rs2282679	C	A	26.85	0.555	1.01	0.882	0.963
*GC*	rs4588	A	C	26.91	0.508	1.00	0.953	0.994
*GC*	rs7041	T	G	43.76	0.445	1.02	0.782	0.963
*LRP2*	rs10210408	C	T	32.98	0.512	0.90	0.165	0.481
*LRP2*	rs11679947	A	G	49.66	1.000	1.12	0.114	0.481
*LRP2*	rs16856596	A	G	27.2	0.884	0.93	0.376	0.779
*LRP2*	rs2239598	C	T	33.46	0.134	1.10	0.185	0.519
*LRP2*	rs2241190	G	A	47.15	0.353	1.13	0.075	0.425
*LRP2*	rs2268373	C	G	24.92	0.643	1.16	0.057	0.425
*LRP2*	rs2544381	C	G	30.8	0.308	1.11	0.160	0.481
*LRP2*	rs3944004	G	T	21.88	0.091	1.23	**0.011**	0.425
*LRP2*	rs4668136	C	T	48.36	0.354	1.10	0.157	0.481
*LRP2*	rs830964	T	C	26.91	0.508	1.03	0.728	0.945
*LRP2*	rs831003	G	C	21.88	0.398	0.87	0.116	0.481
*LRP2*	rs990626	C	T	25.52	0.648	1.14	0.085	0.425
*NADSYN1*	rs3829251	A	G	15.67	0.513	1.07	0.493	0.867
*NADSYN1*	rs7944926	A	G	27.89	0.061	0.96	0.632	0.890
*RXRA*	rs12004589	T	G	12.49	0.426	0.89	0.292	0.687
*RXRA*	rs3118523	G	A	19.57	0.141	1.10	0.268	0.648
*RXRA*	rs3132299	G	C	18.61	0.389	1.01	0.887	0.963
*RXRA*	rs4842196	C	A	25.98	0.940	1.03	0.703	0.945
*RXRA*	rs7864987	C	T	25.55	0.148	1.05	0.508	0.867
*RXRA*	rs9409929	A	G	34.7	0.307	0.90	0.166	0.481
*VDR*	rs11168275	G	A	26.03	0.409	0.94	0.436	0.809
*VDR*	rs11568820	A	G	21.39	0.143	1.11	0.195	0.519
*VDR*	rs11574143	A	G	10.1	0.524	1.07	0.549	0.889
*VDR*	rs12721364	T	C	15.05	0.428	1.04	0.718	0.945
*VDR*	rs1544410	A	G	39.56	0.628	0.96	0.575	0.890
*VDR*	rs1989969	T	C	37.89	0.006	1.01	0.882	0.963
*VDR*	rs2107301	T	C	29.72	0.446	1.01	0.879	0.963
*VDR*	rs2189480	A	C	38.89	0.429	0.89	0.088	0.425
*VDR*	rs2228570	T	C	39.31	0.585	1.02	0.757	0.963
*VDR*	rs2238135	C	G	25.73	0.019	0.99	0.945	0.994
*VDR*	rs2238136	A	G	26.75	0.002	0.96	0.595	0.890
*VDR*	rs2239182	G	A	49.71	0.643	0.96	0.527	0.882
*VDR*	rs2283342	C	T	17.37	0.312	0.91	0.315	0.703
*VDR*	rs2853564	C	T	38.18	0.037	1.01	0.869	0.963
*VDR*	rs4237855	G	A	39.24	0.128	0.88	0.068	0.425
*VDR*	rs4334089	A	G	27.33	0.771	0.99	0.870	0.963
*VDR*	rs7299460	T	C	30.62	0.495	1.10	0.198	0.519
*VDR*	rs731236	C	T	39.01	0.429	0.95	0.428	0.809
*VDR*	rs7970314	G	A	23.11	0.142	1.15	0.087	0.425
*VDR*	rs7975232	C	A	47.35	0.684	1.00	0.979	0.994

a MAF and HWE were calculated among controls only

b Five SNPs (rs11023374, rs1989969, rs2238136, rs2238135, rs2853564) showed significant departure from HWE and were excluded from subsequent analyses

c Odds ratios were estimated using logistic regression adjusted for age and sex and assuming a log additive model for each SNP.

d Adjusted for multiple comparisons using the False Discovery Rate (FDR).

Results are shown in table 3 for the analysis using a log-additive model. Using a log-additive model, several SNPs in CYP24A1 (rs4809958, rs6013905, and rs6127119) and CYP2R1 (rs10741657, rs12794714, and rs2060793) and one SNP in both CASR (rs3804592) and LRP2 (rs3944004) were associated with significant alterations in pancreas cancer risk (Table 3); the unadjusted p-values for these associations ranged from 0.011 to 0.050. After adjustment for multiple comparisons, none of the associations were statistically significant at p<0.05.

Table 4 presents the results of the analysis by genotype categories, not assuming an additive model, for each SNP where at least one genotype (heterozygote or minor homozygote) had a 95% CI that did not overlap 1.0. When analyzed by genotype there were significant associations for 23 of the 87 SNPs evaluated, more than would be expected by chance alone at p<0.05 even considering that a few of the SNPs are in LD. Also, the involvement of 7 out of 11 genes, which presumably are independent, is higher than what would be expected by chance. These associations occurred in the *CASR, CYP24A1, CYP2R1, GC, LRP2, RXRA*, and *VDR* genes. Consistent with the findings from the log-additive models, several SNPs in *CYP24A1* and *CYP2R1* were significantly associated with pancreas cancer risk, including *CYP24A1* rs6127119 (TT versus CC. OR = 1.94; 95%CI: 1.28–2.94) and *CYP2R1* rs10741657 (AA versus GG, OR = 0.70; 95%CI: 0.51–0.95). Four SNPs in LRP2, including rs3944004 (GG versus TT, OR = 1.93; 95% CI: 1.25–2.97), were also significantly associated with risk. No significant associations were observed in the *CUBN, CYP27B1, DHCR7*, and *NADSYN1* genes.

**Table 4:** Age and sex adjusted odds ratios for the associations between SNPs in vitamin D-related genes and pancreas cancer risk among Ontario cases (n = 628) and controls (n = 1193) by genotype categories; results are shown for all SNPs with 95% confidence intervals that do not overlap 1.0.

Gene	SNP	Genotype	Cases N (%)	Control N (%)	Age and Sex adjusted OR	Lower 95% CI	Upper 95% CI
*CASR*	rs12485716	GG	358 (57)	613 (51)	1.00		
		AG	217 (35)	499 (42)	0.74	0.60	0.91
		AA	52 (8)	81 (7)	1.08	0.74	1.57
	rs3804592	GG	491 (78)	869 (73)	1.00		
		AG	124 (20)	306 (26)	0.71	0.56	0.90
		AA	13 (2)	18 (2)	1.32	0.64	2.71
	rs4678174	TT	330 (53)	548 (46)	1.00		
		CT	230 (37)	542 (45)	0.65	0.46	0.92
		CC	68 (11)	103 (9)	0.93	0.66	1.29
*CYP24A1*	rs2181874	GG	338 (54)	665 (58)	1.00		
		AG	239 (38)	460 (39)	1.02	0.83	1.26
		AA	51 (8)	68 (6)	1.48	1.01	2.18
	rs2585428	GG	212 (340	346 (29)	1.00		
		AG	278 (44)	578 (49)	0.79	0.63	0.98
		AA	137 (22)	264 (22)	0.85	0.65	1.11
	rs4809957	GG	362 (58)	749 (63)	1.00		
		GA	232 (37)	377 (32)	1.27	1.04	1.57
		GG	33 (5)	63 (5)	1.11	0.72	1.73
	rs4809958	TT	428 (68)	850 (71)	1.00		
		GT	174 (28)	324 (27)	1.07	0.86	1.33
		GG	26 (4)	19 (2)	2.73	1.49	4.99
	rs6013905^a^	TT	429 (68)	848 (71)	1.00		
		TC	173 (27)	325 (27)	1.05	0.85	1.31
		CC	26 (4)	20 (2)	2.58	1.42	4.68
	rs6097805	AA	325 (52)	680 (57)	1.00		
		GA	258 (41)	428 (36)	1.26	1.03	1.55
		GG	44 (7)	83 (7)	1.13	0.77	1.67
	rs6127119	CC	356 (57)	711 (60)	1.00		
		CT	222 (36)	428 (36)	1.04	0.85	1.28
		TT	48 (8)	50 (4)	1.94	1.28	2.94
*CYP2R1*	rs10741657^b^	GG	262 (42)	451 (38)	1.00		
		AG	286 (46)	550 (46)	0.90	0.73	1.11
		AA	77 (12)	190 (16)	0.70	0.51	0.95
	rs12794714	GG	180 (29)	399 (34)	1.00		
		GA	307 (49)	559 (47)	1.22	0.97	1.53
		AA	141 (23)	234 (20)	1.33	1.01	1.75
*GC*	rs1491711	GG	240 (38)	526 (44)	1.00		
		CG	312 (49)	510 (43)	1.34	1.09	1.65
		CC	76 (12)	155 (13)	1.07	0.78	1.46
*LRP2*	rs11679947	AA	182 (29)	294 (25)	1.00		
		AG	294 (47)	597 (50)	0.80	0.63	1.00
		GG	151 (24)	302 (25)	0.81	0.62	1.06
	rs2268373	GG	315 (50)	675 (57)	1.00		
		CG	273 (44)	440 (37)	1.33	1.08	1.62
		CC	38 (6)	77 (7)	1.04	0.69	1.58
	rs2544381	GG	274 (44)	579 (49)	1.00		
		GC	291 (46)	493 (41)	1.24	1.01	1.53
		CC	62 (10)	121 (10)	1.08	0.77	1.52
	rs3944004	TT	351 (56)	718 (60)	1.00		
		GT	233 (37)	428 (36)	1.11	0.91	1.37
		GG	44 (7)	47 (4)	1.93	1.25	2.97
*RXRA*	rs3118523	AA	382 (61)	780 (65)	1.00		
		AG	226 (36)	359 (30)	1.28	1.04	1.57
		GG	20 (3)	54 (5)	0.75	0.44	1.28
	rs7864987	TT	350 (56)	651 (55)	1.00		
		CT	222 (35)	473 (40)	0.87	0.71	1.07
		CC	56 (9)	68 (6)	1.53	1.05	2.23
*VDR*	rs12721364	CC	454 (73)	854 (72)	1.00		
		CT	150 (24)	312 (26)	0.90	0.72	1.13
		TT	22 (4)	23 (2)	1.81	1.00	3.29
	rs2189480	CC	270 (43)	452 (38)	1.00		
		CA	265 (42)	554 (46)	0.80	0.65	0.99
		AA	93 (15)	187 (16)	0.83	0.62	1.11
	rs4237855	AA	258 (41%)	450 (38%)	1.00		
		AG	287 (46%)	540 (46%)	0.93	0.75	1.15
		GG	83 (13%)	195 (16%)	0.74	0.55	1.00

a rs6013905 is in high linkage disequilibrium with rs4809958 (r^2^ = 0.94).

b rs2060793 is in complete linkage disequilibrium with SNP rs2060793 (r^2^ = 1.00); both SNPs were genotyped in our study and results were the same.

## Discussion

The findings of this study suggest that polymorphisms in vitamin D related pathway genes may be associated with pancreas cancer risk. It has been widely hypothesized that vitamin D may reduce cancer risk, although based on recent published data the association between vitamin D and pancreatic cancer risk is unclear [27]. Findings from a large pooled study of 25(OH)D and pancreas cancer risk have raised the concern that high levels of vitamin D may be associated with increased pancreas cancer risk [12]; however, inverse associations have been observed in a subsequent pooled study of 25(OH)D [13] and some studies of dietary vitamin D intake [14] and predicted vitamin D status [16], [17]. Despite these conflicting findings, no previous studies have evaluated the association between genetic variants that may influence 25(OH)D levels and pancreas cancer risk.

Although none of our associations were significant after adjustment for multiple comparisons, minor homozygotes in several SNPs in the *CYP2R1* gene, including rs10741657, rs2060793, rs12794714, were associated with a 20–30% change in pancreas cancer risk. These CYP2R1 SNPs were significantly associated with 25(OH)D levels in two independent GWAS [18], [19]. Consistent with the vitamin D cancer prevention hypothesis, the minor homozygote of rs10741657 associated with increased 25(OH)D levels and reduced risk of 25(OH)D insufficiency [18], was associated with reduced pancreas cancer risk in this study (AA versus GG, OR = 0.70; 95%CI: 0.51–0.95). Seven of the 18 SNPs evaluated in the *CYP24A1* gene, involved in the breakdown of 25(OH)D and the active 1,25-dihydroxyvitamin D, were associated with pancreas cancer risk. For example, *CYP24A1* rs6127119 TT versus CC genotype was positively associated with pancreas cancer risk (OR = 1.94; 95%CI: 1.28–2.94). A few SNPs in the vitamin D receptor gene were associated with pancreas cancer risk; however, all were borderline significant. The heterozygote of one *GC* (vitamin D binding protein) SNP was associated with pancreas cancer risk.

Other genes with polymorphisms significantly associated with pancreas cancer risk included *LRP2, CASR*, and *RXRA*. The *LRP2* (megalin) gene is involved in cell uptake of vitamins [28], including vitamin D, and variants in *LRP2* have previously been associated with prostate cancer risk [29]; although this mechanism may be due to modified uptake of androgens [29]. The *LRP2* rs3944004 minor homozygote was significantly associated with an almost doubling in pancreatic cancer risk (GG versus TT, OR = 1.93; 95% 1.25–2.97). *LRP2* knockout mice exhibit vitamin D and estrogen deficiency [30] but no studies have evaluated if *LRP2* modifies the association between vitamin D and pancreas cancer. When analyzed by genotype, a few SNPs in *CASR* and *RXRA* were associated with pancreas cancer risk; however, only one was significant in the log-additive models. Polymorphisms in the calcium sensing receptor gene [19], [31],[32] and RXRA [33] have been associated with colorectal cancer risk, but to our knowledge no previous studies have evaluated these genes in relation to pancreas cancer risk.

We have presented the results of our study analyzed using both log additive model and by genotype categories. It is unknown if the biologic effects of the SNPs studied follow an additive model and it is therefore uncertain if this constraint is appropriate; whereas the general genotype model does not make any assumptions about the mode of inheritance. When analysed by genotype, significant odds were observed for heterozygotes of several variants but not for the minor allele homozygotes. These findings suggest that an additive model may not be a good fit for all variants. While it is possible that there is a biologic effect of heterozygotes, it seems likely that small numbers among minor allele homozygotes observed in many variants limited our ability to detect significant associations in these genotypes.

The mechanism by which variability in vitamin D genes may influence pancreatic cancer risk is uncertain. The variants may modify risk of pancreatic cancer through modulation of serum vitamin D levels or by altering cellular activity of vitamin D. Laboratory studies have shown that vitamin D arrests tumor cell growth and induces apoptosis in addition to other chemopreventive mechanisms [8]. The influence of environment-derived vitamin D on these findings cannot be excluded and larger studies will be needed to consider gene-environment interactions. Although we present the results of several novel associations, we cannot rule out the possibility that some of these associations may be due to chance, and the possibility of genetic pleiotropy and linkage disequilibrium. Future studies are needed with a larger sample size to confirm the results that we present here. Furthermore, we were unable to adjust for genetic ancestry in our study and although our results did not change substantially when non-Caucasians were excluded, future studies should further consider the possibility of population stratification.

This study is the first to evaluate associations between genes involved in the vitamin D pathway and pancreas cancer risk. Our findings suggest that genes known to be associated with 25(OH)D are associated with pancreas cancer risk and that polymorphisms in several genes involved in vitamin D activity may be associated with pancreas cancer risk. It is possible that the latter genetic variants modify the association between 25(OH)D and pancreas cancer risk and gene-environment interactions may explain the previous inconsistent findings. Variants in vitamin D-related genes may influence pancreatic cancer risk by modifying long-term serum 25(OH)D levels or by altering expression levels in vitamin D-responsive genes through alterations in the cellular activity of vitamin D. Several common cancers have shown increased risk with low vitamin D levels, leading many to suggest vitamin D supplementation as strategy to reduce the risk of these malignancies. With the recent suggestion that high vitamin D levels may be associated with increased pancreatic cancer risk, the elucidation of the genetic influences of vitamin D activity and pancreatic cancer may be crucial in understanding this association and may have important implications on cancer-prevention strategies centered on vitamin D.
